# Imaging microtubule dynamics: A new frontier in biomarker development for neurodegenerative diseases

**DOI:** 10.1002/alz.70670

**Published:** 2025-09-23

**Authors:** Naresh Damuka, Samuel N. Lockhart, Kiran K. Solingapuram Sai

**Affiliations:** ^1^ Department of Radiology Wake Forest School of Medicine Winston‐Salem North Carolina USA; ^2^ Department of Gerontology and Geriatric Medicine Wake Forest School of Medicine Winston‐Salem North Carolina USA

**Keywords:** [^11^C]MPC‐6827, Alzheimer's disease, diagnosis, microtubules, neurodegeneration, PET imaging

## Abstract

Microtubules (MTs) are essential components of the neuronal cytoskeleton, playing key roles in intracellular transport, synaptic function, and overall neuronal integrity. Although MT dynamics and MT‐binding agents have been studied extensively, their potential as biomarkers in neurodegenerative diseases has received limited attention. Emerging evidence suggests that MT destabilization is one of the earliest pathological events in Alzheimer's disease, Parkinson's disease, and other related disorders. This review highlights MT dysregulation as a promising marker of early neurodegenerative changes and discusses recent advances in imaging tools, particularly positron emission tomography (PET), that enable in vivo visualization of MT dynamics. We focus on the development and application of novel MT‐targeting PET radiotracers, such as [^11^C]MPC‐6827, which demonstrate high specificity for destabilized MTs and excellent brain uptake. To our knowledge, this is the first comprehensive review emphasizing MT alterations as a translational imaging biomarker, offering a new perspective in the early detection and monitoring of neurodegenerative diseases.

**Highlights:**

- Microtubule (MT) instability is an early and underrecognized event in neurodegenerative disease pathogenesis and may precede classical hallmarks of Alzheimer's disease pathology.
- MT dysregulation holds promise as a novel diagnostic biomarker, offering new opportunities for early detection and disease monitoring in Alzheimer's disease, Parkinson's disease, and related disorders.
- Recent advances in MT‐targeted positron emission tomography imaging, particularly with [^11^C]MPC‐6827, enable non‐invasive, in vivo visualization of MT dynamics with high specificity and brain penetration.
- Cross‐species validation of MT imaging, from rodent models to non‐human primates and humans, demonstrates strong translational potential, supporting its future clinical application.
- Integration of MT imaging with established amyloid, tau, and neuroinflammation markers enhances diagnostic precision, supports early intervention strategies, and enables more personalized approaches to neurodegenerative disease care.

## 1 INTRODUCTION

Neurodegenerative disorders, including Alzheimer's disease (AD),^1^ Parkinson's disease (PD),^2^ and amyotrophic lateral sclerosis (ALS),^3^ are characterized by the progressive deterioration of neuronal structure and function, leading to cognitive and motor impairments.^4^ Despite extensive research efforts, the underlying molecular mechanisms driving these diseases remain incompletely understood, and reliable biomarkers for early diagnosis are still lacking. Over recent years, growing evidence has highlighted the crucial role of cytoskeletal dysfunction, particularly involving microtubules (MTs), in the pathogenesis of neurodegenerative disorders.^5^

**FIGURE 1:**
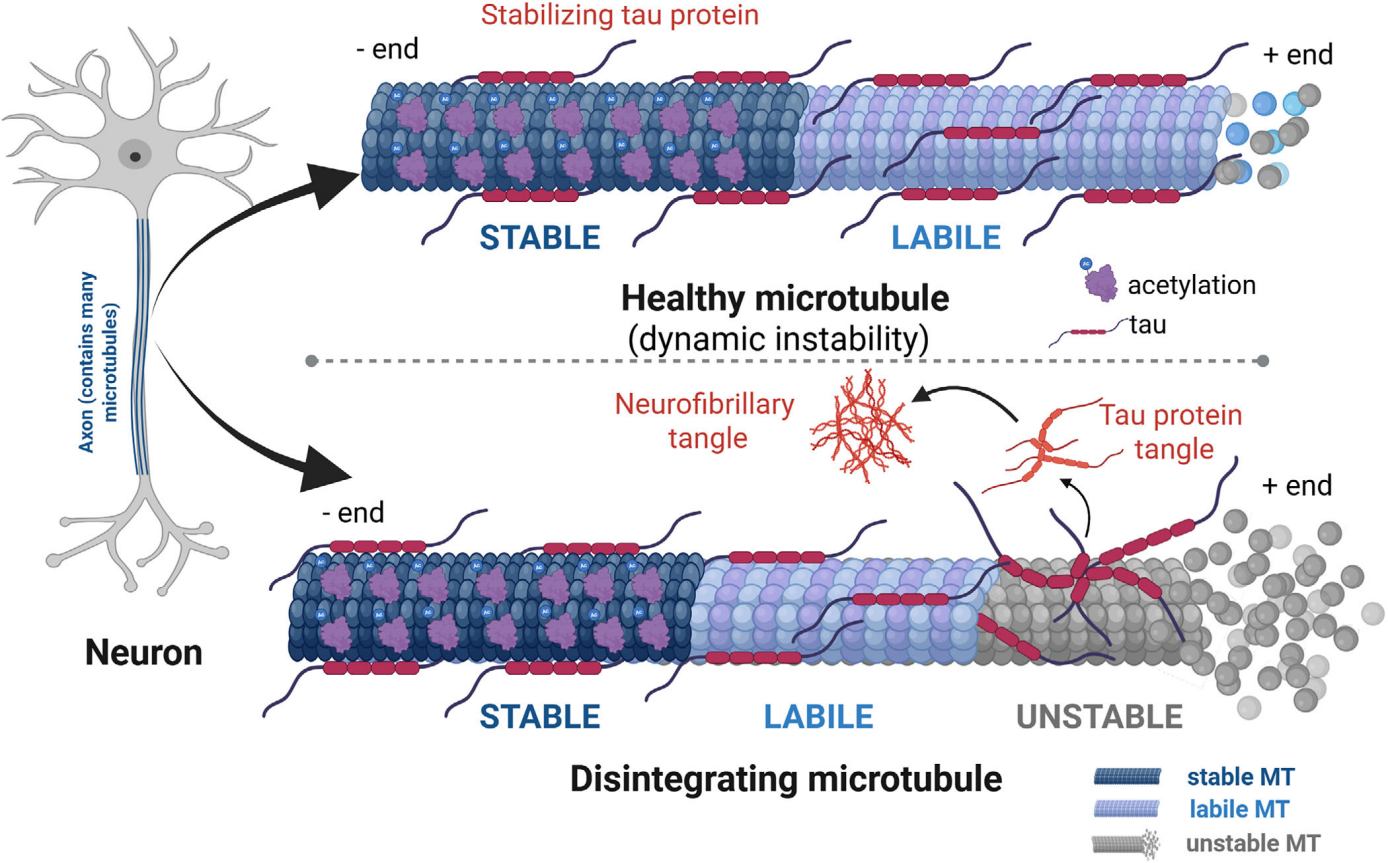
Schematic representation of healthy and disintegrating microtubules (MTs) in neurons. This figure illustrates the structural and molecular differences between healthy and degenerating MTs within neurons. The neuron diagram on the left highlights axons, which contain numerous MTs essential for neuronal function. The upper panel depicts a healthy MT composed of both stable (dark blue) and labile (light blue) domains, organized in a hollow cylindrical structure. Tau proteins are shown stabilizing the MT lattice, with post‐translational modifications such as acetylation contributing to MT integrity. In this state, unstable MTs (gray) are minimal, maintaining axonal transport and neuronal health. In contrast, the lower panel illustrates a disintegrating MT associated with neurodegeneration. Although some stable and labile MT regions remain, there is a marked increase in unstable MTs (gray) leading to breakdown of MT structure. Detachment and aggregation of tau proteins contribute to the formation of tau tangles and neurofibrillary tangles, pathological hallmarks of diseases such as Alzheimer's disease. This visual emphasizes MT destabilization as an early event in neurodegenerative pathology. Figure created with BioRender.com.

MTs are highly dynamic cytoskeletal components essential for maintaining cellular architecture, intracellular transport, and mitotic processes.^6^ In neurons, their stability and dynamic remodeling are fundamental for axonal transport,^7^ synaptic function, and plasticity.^8^ However, in neurodegenerative conditions, MT dynamics become dysregulated, leading to destabilization, impaired transport, and neuronal dysfunction.^9^ Tau protein, which plays a critical role in stabilizing MTs, becomes hyperphosphorylated in diseases such as AD, contributing to MT disassembly and aggregation into neurofibrillary tangles (NFTs) ^10^ (Figure 1). Similarly, in PD and ALS, MT dysfunction exacerbates neuronal degeneration by disrupting intracellular trafficking and structural integrity.^5^, ^11^ These findings underscore the importance of MT stability in maintaining neuronal health and suggest that MTs may serve as a promising biomarker and potential therapeutic target across diverse neurodegenerative conditions.^12^

Recent advancements in molecular imaging, particularly positron emission tomography (PET), have facilitated the visualization and quantification of MT dynamics in vivo.^13^, ^14^ Radiotracers such as [^11^C]MPC‐6827, which selectively bind to destabilized MTs, offer promising avenues for early detection and disease monitoring.^14^ These imaging tools provide unprecedented insights into the progression of neurodegenerative diseases and the potential efficacy of therapeutic interventions aimed at restoring MT stability.

This review aims to comprehensively explore the role of MT instability in neurodegeneration and its potential as a translational biomarker. The subsequent sections will delve into the structure and dynamic instability of MTs, their divergent behaviors in neuronal versus non‐neuronal cells, and the functional implications of their dysregulation in neurodegenerative diseases. In addition, we discuss therapeutic strategies targeting MT stabilization and evolving methodologies, including PET imaging, that are revolutionizing our understanding of MT dysfunction in neurodegeneration.

## 2 OVERVIEW OF MT STRUCTURE AND DYNAMIC INSTABILITY

MTs, essential components of the eukaryotic cytoskeleton, are dynamic cylindrical polymers composed of α‐ and β‐tubulin dimers. Their structural organization and behavior underpin diverse cellular processes, including intracellular transport, cell shape maintenance, and mitotic spindle assembly.^15^ The unique ability of MTs to switch rapidly between phases of growth and shrinkage, a phenomenon known as dynamic instability, is fundamental to their biological roles and adaptability within the cell.^16^, ^17^

MTs are hollow tubes, ≈25 nm in diameter, typically formed by 13 protofilaments aligned in a parallel manner along their longitudinal axis.^18^ Each protofilament is a linear chain of head‐to‐tail aligned α‐ and β‐tubulin heterodimers, creating intrinsic structural polarity. The plus end, characterized by exposed β‐tubulin, is highly dynamic, favoring the addition and removal of tubulin subunits, whereas the minus end, with exposed α‐tubulin, is generally more stable and anchored at microtubule‐organizing centers (MTOCs), such as the centrosome.^19^

Tubulin polymerization is driven by the binding of guanosine triphosphate (GTP) to β‐tubulin, which promotes stability and lattice assembly. Upon incorporation into the MT lattice, β‐tubulin undergoes GTP hydrolysis, converting GTP to guanosine diphosphate (GDP), which destabilizes the lattice and primes the MT for depolymerization. This dual role of GTP, stabilizing polymerization at the growing ends and triggering instability upon hydrolysis, lies at the heart of MT dynamics.^20^

Dynamic instability, the hallmark of MT, reflects their ability to alternate between growth and shrinkage.^21^ During polymerization, GTP‐bound tubulin dimers add to the plus end, forming a stabilizing GTP cap that prevents depolymerization and maintains structural integrity. The loss of this GTP cap due to GTP hydrolysis marks the onset of catastrophe, a rapid depolymerization phase characterized by protofilament peeling and MT shrinkage. However, MTs can recover through rescue events, wherein remnant patches of GTP‐bound tubulin within the lattice nucleate new growth, restoring the GTP cap and enabling reassembly.^20^

Cryo‐electron microscopy (cryo‐EM) has provided detailed insights into the structural transitions underlying dynamic instability. The curved‐to‐straight conformational change of tubulin dimers during polymerization, coupled with the transition to GDP‐bound states, reveals the molecular basis of MT instability.^22^ High‐resolution cryo‐EM studies have elucidated lateral interactions between protofilaments, and the structural variations induced by nucleotide state changes. It is intriguing that the seam within the MT lattice, where α‐tubulin and β‐tubulin form heterotypic lateral interactions, has emerged as a potential weak point that influences disassembly.^19^, ^23^, ^24^

GTP hydrolysis–induced lattice compaction, observed through structural studies, highlights the interplay between nucleotide states and MT stability. The binding of stabilizing agents like paclitaxel has been shown to counteract the effects of GTP hydrolysis, underscoring the therapeutic potential of targeting these molecular mechanisms.^20^ Moreover, the discovery of MT plus‐end tracking proteins, such as EB3, has advanced our understanding of how protein interactions modulate growth and shrinkage dynamics.^25^ Connecting these foundational insights, the next section will contrast the distinct MT dynamics observed in neurons compared to non‐neuronal cells, highlighting the specialized structural and functional requirements that shape their behavior.

## 3 NEURONAL VERSUS NON‐NEURONAL MT DYNAMICS

MTs are essential components of the cytoskeleton across diverse cell types, but their stability and dynamics vary significantly depending on cellular context. This is particularly evident in neurons, where MTs must support long axons and dendrites, enabling efficient cellular transport and signaling.^15^ Neuronal MTs are highly polarized, with axons exhibiting a uniform plus‐end‐out orientation, whereas dendrites display mixed polarity.^26^ This organization, along with motor proteins such as kinesins and dyneins, supports bidirectional transport of organelles and signaling molecules critical for neuronal function.^27^

Unlike MTs in proliferating cells, which exhibit high dynamic instability to accommodate rapid cytoskeletal remodeling during division and migration, neuronal MTs are relatively stable. This stability is enhanced by extensive post‐translational modifications (PTMs) such as acetylation, detyrosination, and polyglutamylation.^28^ These modifications modulate interactions with microtubule‐associated proteins (MAPs) and contribute to reduced dynamic behavior. For example, acetylated α‐tubulin supports MT longevity and axonal integrity. Neurons also express specific stabilizing MAPs like tau and MAP2, which differ from the more dynamic MAPs (e.g., stathmin) found in non‐neuronal cells.^29^

Molecular factors further contribute to this divergence. For example, the presence of a persistent GTP cap, a stabilizing structure formed by GTP‐bound tubulin subunits at the growing end of MTs in neuronal MTs, helps delay catastrophe events, which are rapid transitions from growth to shrinkage. This supports long‐term stability, whereas non‐neuronal MTs lose this cap more readily, leading to more frequent shrinkage occurrences.^30^, ^31^ Cryo‐EM studies reveal that structural distinctions neuronal MTs have more stable lateral protofilament interactions and fewer lattice defects than their non‐neuronal counterparts. These features help maintain neuronal architecture over long periods.^32^

Despite their relative stability, neuronal MTs are not static. Localized dynamic instability remains crucial for processes such as synaptic plasticity, axonal remodeling, and response to injury.^33^, ^34^, ^35^ The delicate balance between stability and flexibility is tightly regulated and often disrupted in neurodegenerative diseases, highlighting the importance of proper MT dynamics for neuronal health.^36^

**FIGURE 2:**
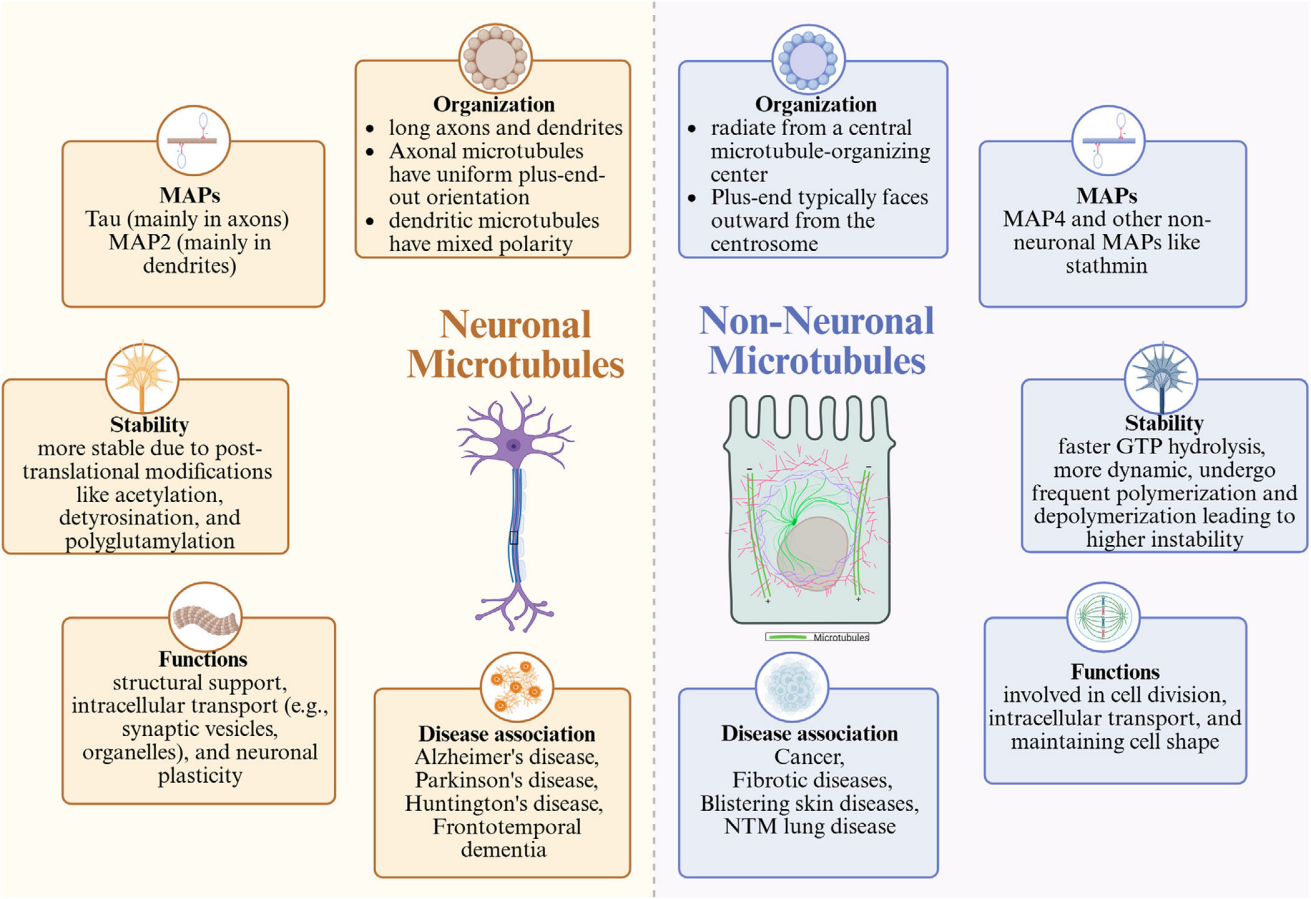
Comparative schematic representation of neuronal versus non‐neuronal microtubules (MTs). This figure contrasts the structural and functional characteristics of MTs in neuronal and non‐neuronal cells. The left panel highlights features of neuronal MTs, which are organized along long axons and dendrites. Axonal MTs exhibit uniform plus‐end‐out polarity, whereas dendritic MTs show mixed polarity. Neuronal MTs are regulated primarily by microtubule‐associated proteins (MAPs) such as tau (in axons) and MAP2 (in dendrites), and are stabilized through post‐translational modifications including acetylation, detyrosination, and polyglutamylation. These MTs support structural integrity, intracellular transport (e.g., synaptic vesicles and organelles), and neuronal plasticity, and are implicated in neurodegenerative diseases such as Alzheimer's disease, Parkinson's disease, Huntington's disease, and frontotemporal dementia (FTD). The right panel depicts non‐neuronal MTs, which are typically arranged in a radial pattern from a central microtubule‐organizing center (MTOC), with plus ends facing outward from the centrosome. These MTs are regulated by MAP4 and other non‐neuronal MAPs such as stathmin, and exhibit faster guanosine triphosphate hydrolysis, higher dynamic instability, and frequent polymerization/depolymerization cycles. They play essential roles in cell division, intracellular transport, and maintenance of cell shape, and are associated with pathologies such as cancer, fibrotic diseases, blistering skin diseases, and nontuberculous mycobacterial lung disease. Figure created with BioRender.com.

In contrast, non‐neuronal cells prioritize flexibility and adaptability.^37^ Their dynamic MT turnover facilitates critical functions such as mitosis, cell migration, and immune surveillance. These differences underscore the specialized requirements of each cell type and the complexity of targeting MT dynamics in disease.^38^ Understanding of these cellular distinctions, aided by advances in structural biology and imaging, is vital for developing therapeutic strategies that address MT‐related pathologies. Figure 2 summarizes the key differences between neuronal and non‐neuronal MTs in structure, function, and regulatory mechanisms.

## 4 FUNCTIONAL SIGNIFICANCE IN NEURONS

In neurons, MTs form the structural backbone for intracellular transport, development, and synaptic communication. They guide motor proteins like kinesins and dyneins, enabling long‐range transport of organelles, messenger RNAs (mRNAs), and vesicle precursors—vital for sustaining neuron function across extended processes.^39^, ^40^ MTs also drive morphogenesis^41^: from neurite initiation to axon specification^42^ and dendritic arborization.^43^ Local MT stabilization underlies early neurite outgrowth, whereas persistent MT stability contributes to axon formation and polarity.^44^ Beyond structure and transport, MTs participate in activity‐dependent remodeling, synaptic function, and signal transduction.^45^, ^46^ Their dynamic nature supports plasticity, whereas their stabilization ensures reliability—together enabling neurons to maintain functionality and adaptability throughout life.^47^

## 5 MT INSTABILITY IN NEURODEGENERATION

MT disruption is a hallmark of neurodegenerative diseases, contributing to the progressive loss of neuronal function and structural integrity.^48^ The dynamic instability of MTs, which enables essential processes such as intracellular transport, synaptic plasticity, and neuronal remodeling, is severely compromised in these disorders.^49^ Each neurodegenerative disease presents a unique interplay of MT‐associated disruptions, driven by molecular defects in MT‐regulating proteins, PTMs, and toxic protein aggregates. The following section delves into the specific MT‐related disruptions observed in conditions such as AD, PD, and others, with a focus on the underlying molecular mechanisms and their relevance for biomarker development and therapeutic targeting.^50^

### 5.1 Alzheimer's disease (AD)

MT disruption is a significant contributor to the pathogenesis of AD, affecting neuronal function and structural integrity. In AD, the relationship between MTs and pathological protein aggregates, particularly tau and amyloid beta (Aβ), has been studied extensively to understand their roles in disease progression.^51^

In AD, tau undergoes abnormal hyperphosphorylation, which reduces its affinity for MTs, leading to their destabilization and disassembly.^52^, ^53^ This destabilization impairs axonal transport, resulting in the accumulation of essential cargo, such as organelles and synaptic vesicles, within axons.^10^ In addition, hyperphosphorylated tau increases the susceptibility of MTs to severing by proteins such as katanin and spastin, leading to further MT fragmentation and loss of neuronal integrity.^54^

Recent evidence suggests that tau may not function primarily as a stabilizer of MTs but rather as a regulator of their dynamic remodeling by interacting with labile domains.^5^ Studies of tau depletion in cultured neurons have demonstrated a reduction in labile MT mass and an increase in stable MT mass, highlighting tau's role in maintaining MT dynamics.^55^ The redistribution of MT‐associated proteins, such as MAP6, in response to tau depletion further underscores the complexity of MT regulation in AD.^53^

In addition to tauopathy, Aβ oligomers have been implicated in MT alterations through distinct mechanisms. Studies have demonstrated that Aβ^42^ induces MT stabilization via the activation of RhoA‐dependent pathways, leading to the formation of detyrosinated, stable MTs in the early stages of AD.^56^, ^57^, ^58^ However, this stabilization is transient and time dependent, peaking ≈1.5 h after Aβ exposure and returning to baseline levels after 8 h.^59^ Of interest, Aβ‐driven stabilization of dynamic MTs is associated with tau hyperphosphorylation and subsequent synaptic dysfunction. Conversely, Aβ oligomers can also induce MT loss by promoting tubulin polyglutamylation and recruiting severing enzymes such as spastin, further contributing to cytoskeletal breakdown and neurodegeneration.^60^, ^61^

Beyond tau‐ or Aβ‐mediated mechanisms, studies have shown that MT loss and dysfunction occur early in AD. Morphometric analyses of pyramidal neurons from patients with AD have revealed a significant reduction in both the number and total length of MTs, independent of tau deposition.^62^ This suggests that MT impairment is an early pathological event in AD, potentially contributing to neuronal dysfunction before NFTs become apparent.

The PTMs of tubulin—including acetylation, tyrosination, and polyglutamylation—play a crucial role in regulating MT stability and function.^63^ In AD, decreased acetylation and increased polyglutamylation have been observed, impairing motor protein interactions and further compromising axonal transport. Notably, hyperphosphorylated tau has been shown to recruit tubulin tyrosine ligase‐like 6 (TTLL6), promoting tubulin polyglutamylation and facilitating MT severing by spastin, and ultimately contributing to MT degradation.^64^, ^65^

### 5.2 Parkinson's disease (PD)

In PD, MT instability is linked to the accumulation of α‐synuclein aggregates, which interact with MTs to alter their dynamics and induce toxicity.^66^ Patient‐derived fibroblasts display reduced MT mass, increased unpolymerized tubulin, and aberrant tubulin PTMs, such as excessive polyglutamylation. These changes promote MT disassembly and disrupt the movement of mitochondria and vesicles, exacerbating oxidative stress and energy deficits in neurons.^67^ Toxins like methyl‐4‐phenylpyridinium (MPP+), known to induce PD‐like pathology, disrupt MT stability before impairing mitochondrial function, highlighting the upstream role of MT dysregulation in PD.^68^, ^69^ The intricate relationship between α‐synuclein and tau also plays a role, as their interactions accelerate aggregation and worsen neurodegenerative outcomes.

### 5.3 Frontotemporal dementia (FTD)

Frontotemporal dementia (FTD), particularly the familial form caused by autosomal dominant mutations in the *MAPT* gene, exemplifies the pathological impact of tau dysregulation on MT dynamics. These mutations lead to tau hyperphosphorylation, mislocalization, and aggregation within the neuronal soma, disrupting tau's normal axonal distribution and MT‐binding function. This aberrant localization impairs MT assembly and stability, alters axonal transport, and contributes to nuclear membrane deformation by affecting MT anchoring at the nuclear envelope.^70^ Recent findings indicate that tau‐mediated disruption of MTs in FTD‐MAPT neurons results in defective nucleocytoplasmic transport, a hallmark shared with other neurodegenerative diseases such as ALS. The accumulation of toxic tau aggregates interferes with proteostasis and autophagy, further exacerbating neuronal stress. These MT‐related abnormalities in FTD not only highlight the functional consequences of tau dysregulation but also point to converging mechanisms of neurodegeneration shared across tauopathies.^71^

### 5.4 Other neurodegenerative diseases

Huntington's disease (HD) and ALS further illustrate the critical role of MT dynamics in neurodegeneration.^72^ In ALS, the loss of stathmin‐2, a protein that promotes MT disassembly, leads to axonal degeneration, reflecting the necessity of balanced MT turnover.^73^ In HD, defective MT‐associated proteins and PTM imbalances impair MT function, disrupting the transport of essential cargo and compounding neuronal stress. These disruptions culminate in the degeneration of motor neurons and cognitive decline observed in ALS and HD patients.^50^

Hereditary spastic paraplegia (HSP) is an inherited, rare neurodegenerative disorder characterized by weakness in the lower extremities and spasticity. HSP provides a unique perspective on the consequences of hyper‐stabilized MTs. Mutations in spastin, a MT‐severing protein, lead to the accumulation of overly stable MTs along axons. This hyper‐stability reduces the dynamic plus‐ends necessary for cargo transport and axonal flexibility, resulting in axonal swellings and degeneration of corticospinal tracts.^60^ Of interest, treatment with MT‐destabilizing agents, such as nocodazole, has shown promise in alleviating these defects, underscoring the importance of maintaining a dynamic equilibrium in MT behavior.^74^

The PTMs of tubulin also play a central role in regulating MT dynamics in neurodegeneration. Acetylation enhances MT stability and motor protein interactions, supporting axonal transport. However, diseases such as AD, PD, and HD are characterized by decreased tubulin acetylation, often due to elevated histone deacetylase 6 (HDAC6) activity.^75^ Conversely, excessive polyglutamylation destabilizes MTs and impairs cargo transport, as seen in PD and other disorders. Detyrosination, a marker of stable MTs, is dysregulated in neurodegenerative diseases, further highlighting the intricate balance required for neuronal health.^76^

Although MT instability is a predominant feature of neurodegeneration, hyper‐stabilization can also be detrimental. Hyper‐stable MTs lack the dynamic remodeling required for processes such as synaptic plasticity and neuronal repair. In healthy neurons, paclitaxel‐induced stabilization enlarges growth cones but reduces neurite extension and axonal transport, illustrating that excessive stability impairs cellular function.^77^ Similarly, hyper‐stable MTs in HSP hinder neuronal function, leading to axonal swellings and degeneration.^78^ These observations underscore the necessity of maintaining MT homeostasis, a finely tuned balance between stability and plasticity to support neuronal integrity, transport, and adaptability.

## 6 THERAPEUTIC STRATEGIES FOR ADDRESSING MT INSTABILITY

MT instability, a hallmark of several neurodegenerative diseases, has driven research into therapeutic strategies aimed at restoring MT dynamics and mitigating associated neuronal dysfunction. These approaches include the use of MT‐stabilizing agents (MSAs), drugs targeting MAPs, and combination therapies that address other neurodegeneration‐associated pathways.^79^

### 6.1 MT‐stabilizing agents

MSAs are a class of compounds that enhance MT polymerization, thereby counteracting the destabilizing effects observed in neurodegenerative conditions. These agents bind to specific sites on the β‐tubulin subunit, such as the taxane or laulimalide/peloruside sites, thereby stabilizing MTs by promoting lateral and longitudinal interactions between protofilaments. Notable MSAs include paclitaxel, epothilone D (EpoD), and laulimalide. Taxane‐site ligands, such as paclitaxel and EpoD, induce a helical conformation of the M‐loop in β‐tubulin, facilitating MT stabilization. Similarly, laulimalide and peloruside A act as molecular “clamps,” strengthening interactions at the MT lattice seam. These mechanisms not only restore MT stability but also improve axonal transport and neuronal integrity in preclinical models of neurodegenerative diseases.^80^, ^81^, ^82^ However, the inability of many MSAs to cross the blood–brain barrier (BBB) limited their utility for treating central nervous system (CNS) diseases.

In contrast, EpoD, a taxane‐derived compound, emerged as a more viable candidate due to its excellent brain penetration. Studies in tau transgenic mouse models demonstrated that EpoD not only stabilized MTs but also reduced tau pathology, improved neuronal integrity, and reversed cognitive deficits. Despite these encouraging results, the dose‐dependent neurotoxicity of EpoD highlighted the delicate balance required to modulate MT dynamics without adversely affecting neuronal and glial cells.^83^, ^84^ Similar challenges have been observed with other MT stabilizers, such as TPI‐287 and davunetide, the clinical trials of which revealed limited efficacy or unwanted side effects, emphasizing the complexity of targeting such a critical cellular system.^85^

### 6.2 Targeting tau pathology

The role of MAPs, particularly tau, in maintaining MT stability has also emerged as a critical therapeutic target. Under normal conditions, tau stabilizes MTs and supports axonal transport.^86^ However, in neurodegenerative diseases, tau undergoes pathological modifications, including hyperphosphorylation, reducing its binding affinity for MTs and leading to their destabilization.^87^ Therapeutic approaches targeting tau include small molecules that inhibit tau aggregation, antisense oligonucleotides (ASOs) that reduce tau expression, and compounds that modulate tau PTMs, such as phosphorylation and acetylation. For example, ASOs like BIIB080 have shown promise in reducing tau levels and slowing neurodegeneration in preclinical and early clinical studies.^88^ Similarly, tau aggregation inhibitors, such as TRx0237, aim to prevent the formation of NFTs, preserving MT stability and neuronal function.^89^

### 6.3 Modulating PTMs

Although tau‐targeted therapies hold great promise, challenges remain. Tau is involved in numerous cellular processes, and reducing its levels indiscriminately may disrupt other critical functions. Therapeutic strategies must therefore distinguish between pathological and physiological forms of tau to avoid unintended consequences. In addition, the heterogeneity of neurodegenerative diseases necessitates a multifaceted approach that addresses the interplay between tau pathology, MT instability, and other cellular dysfunctions. PTMs of tubulin and tau add another layer of complexity to MT dynamics and present opportunities for therapeutic intervention. Acetylation, one of the key PTMs, enhances MT stability, and its disruption has been implicated in neurodegenerative diseases. Inhibitors of deacetylating enzymes such as HDAC6 and Sirt2 have shown promise in restoring tubulin acetylation, improving axonal transport, and exerting neuroprotective effects in models of AD, PD, and HSP.^90^ Compounds like tubastatin A, a selective HDAC6 inhibitor, have reversed behavioral and cognitive deficits in tauopathy models, highlighting their therapeutic potential.^91^ However, the pleiotropic roles of HDAC6 and other enzymes necessitate careful evaluation to minimize off‐target effects.^92^

Beyond acetylation, other PTMs such as phosphorylation and O‐GlcNAcylation significantly influence MT and tau function. The inhibition of kinases like glycogen synthase kinase 3 beta (GSK‐3β), which phosphorylates tau at multiple sites, has been explored to reduce pathological tau modifications.^93^ Although compounds such as tideglusib demonstrated efficacy in preclinical models, their limited success in clinical trials underscored the challenges of selectively targeting PTMs without disrupting other essential cellular processes. Similarly, modulating O‐GlcNAcylation, which can prevent tau phosphorylation by blocking key sites, offers a novel therapeutic approach. Early studies with O‐GlcNAcase inhibitors, such as ceperognastat, showed up to a 50% reduction in tau aggregation and a slowing of neurodegeneration in preclinical tauopathy models.^94^ However, these benefits failed to translate into clinical improvement in AD patients during a Phase 2 trial.

### 6.4 Immunotherapies and MT stabilization

Immunotherapy targeting tau and amyloid proteins has emerged as a promising avenue for addressing MT instability in neurodegenerative diseases. Several active and passive immunotherapy strategies have been developed to mitigate tau pathology, which indirectly affects MT stability by reducing tau's pathological gain of function. Active immunizations with peptides like AADvac1, derived from the mid‐domain of tau involved in MT binding, aim to prevent tau aggregation and stabilize MTs by maintaining tau's physiological role.^95^, ^96^ Similarly, ACI‐35 targets a phosphoepitope of tau, potentially mitigating hyperphosphorylation and its destabilizing effects on MTs.^97^

Passive immunotherapies with monoclonal antibodies (mAbs) have shown considerable potential. Antibodies such as BIIB076, Bepranemab, and E2814 target the middle domain of tau, whereas others like Gosuranemab and Semorinemab focus on the N‐terminal region implicated in tau spreading. Additional antibodies, including JNJ63733657 and Lu AF87908, are designed to neutralize neurotoxic phosphorylated tau species.^98^, ^99^, ^100^ These approaches aim to reduce tau levels or interfere with tau aggregation, indirectly preserving MT integrity. However, some trials have faced setbacks due to side effects, such as RG7345, or failure to meet clinical endpoints, as with BIIB076, highlighting the challenges of achieving efficacy while minimizing risks.^101^

mAbs rely on passive diffusion across the BBB, resulting in low CNS penetrance (≈0.1%–0.2% of the injected dose). Despite this, some mAbs can access intracellular tau pools, and this appears to significantly influence their therapeutic effectiveness.^102^ Although certain antibodies can be internalized, others remain confined to the extracellular space, where tau concentrations are lower. Factors such as charge and dissociation constants determine neuronal uptake, posing challenges for the consistent delivery of therapeutic effects. Despite these limitations, reducing tau levels through immunotherapy remains a promising strategy, especially given the association between elevated tau levels and MT instability in AD and other tauopathies.^103^

In addition to tau, targeting Aβ with mAbs has also shown potential for mitigating MT instability. Amyloid plaques can exacerbate tau pathology and MT destabilization through secondary mechanisms. By clearing Aβ aggregates, therapies like lecanemab or donanemab may indirectly influence tau phosphorylation and aggregation, preserving MT dynamics. Although these therapies are in the early stages of exploration for their effects on MT‐related processes, their potential to synergistically address amyloid and tau pathology positions them as valuable components of a broader therapeutic strategy. The integration of amyloid and tau immunotherapies into MT targeting paradigms offers a multifaceted approach to counteracting the cascading effects of neurodegeneration on cellular stability.^104^, ^105^, ^106^, ^107^ A summary of key therapeutic compounds and their mechanisms targeting MT instability is provided in Table 1.

**TABLE 1:** Summary of therapeutic compounds targeting MT instability and related pathways in neurodegenerative diseases.

Compound	Target	Mechanism of action	Clinical status	Clinical population	Effect on MT stability	Ref
**1. Microtubule‐stabilizing agents (MSAs)**						
Paclitaxel	β‐tubulin (taxane site)	Promotes polymerization and lateral protofilament stability	Preclinical	Healthy mice (C57Bl/6)	High (in vitro)	^81^
Epothilone D (EpoD)	β‐tubulin (taxane site)	MT stabilization; reduces tau pathology	Phase I/II (halted)	Tau transgenic mice; NSCLC patients	High	^84^, ^108^
Laulimalide	β‐tubulin (laulimalide site)	Stabilizes MT seam	Preclinical	In vitro/cell models	Moderate	^82^
TPI‐287	β‐tubulin	MT stabilization	Phase I/II	AD, PSP, CBD; neuroblastoma/medulloblastoma	Moderate	^85^
Davunetide	Tau peptide mimic	Enhances MT binding	Phase II (failed)	Prodromal AD, MCI	Moderate	^109^, ^110^
**2. Targeting tau expression and aggregation**						
BIIB080	Tau mRNA	ASO to reduce tau expression	Phase I ongoing	Mild AD	Indirect (via tau reduction)	^88^, ^111^
TRx0237 (LMTX)	Tau aggregates	Inhibits tau aggregation	Phase III (inconclusive)	Mild to moderate AD	Indirect	^89^
**3. Modulation of PTMs**						
Tubastatin A	HDAC6	Increases tubulin acetylation	Preclinical	AD transgenic mice	High	^92^
Tideglusib	GSK‐3β	Reduces tau phosphorylation	Phase II (limited efficacy)	Mild to moderate AD	Indirect	^93^
**4. Tau immunotherapy**						
AADvac1	Tau mid‐domain	Active vaccine against aggregated tau	Phase II ongoing	Mild to moderate AD	Indirect	^112^, ^113^
ACI‐35	Phospho‐tau	Vaccine targeting tau phospho‐epitope	Phase I/II	Early AD	Indirect	^114^
Gosuranemab, Semorinemab, Zagotenemab	N‐terminal tau	Passive immunotherapy—mAbs	Phase II/III	Early to moderate AD	Indirect	^95^, ^96^, ^97^, ^115^
BIIB076, Bepranemab, E2814	Middle‐domain tau	Passive immunotherapy—mAbs	Phase II/III	Prodromal to mild AD; familial AD	Indirect	^99^, ^100^, ^101^
JNJ63733657, Lu AF87908	Phospho‐tau	Passive immunotherapy—mAbs	Phase I/II	Healthy and mild AD	Indirect	^98^, ^116^
**5. Amyloid beta (Aβ) immunotherapy**						
Aducanumab	Aβ plaques	Reduces amyloid load, indirectly stabilizes MTs via tau modulation	FDA Approved ‐2021 (withdrawn 2024)	Early AD	Indirect	^104^, ^117^
Lecanemab	Soluble Aβ protofibrils	Clears soluble Aβ; reduces tau spread	FDA approved (2023)	Early AD	Indirect	^105^, ^118^
Donanemab	Aβ plaques (modified Aβ epitope)	Clears Aβ plaques; slows cognitive decline	FDA approved (2024)	Early symptomatic AD	Indirect	^106^, ^119^
Gantenerumab	Aβ plaques	Binds aggregated Aβ; promotes clearance	Phase III (failed to meet endpoint)	Mild AD	Indirect	^107^, ^120^
Solanezumab	Soluble Aβ	Targets monomeric Aβ; reduces accumulation	Phase III (no significant benefit)	Preclinical AD (DIAN‐TU)	Indirect	^121^, ^122^
Remternetug	Aβ plaques (modified Aβ epitope)	Clears Aβ plaques; slows cognitive decline	Phase III	Early AD	Indirect	^123^

Abbreviations: Aβ, amyloid‐beta; AD, Alzheimer’s disease; ASO, antisense oligonucleotide; CBD, corticobasal degeneration; DIAN‐TU, Dominantly Inherited Alzheimer Network–Trial Unit; FDA, U.S. Food and Drug Administration; GSK‐3β, glycogen synthase kinase‐3 beta; HDAC6, histone deacetylase 6; mAb, monoclonal antibody; MCI, mild cognitive impairment; MT, microtubule; NSCLC, non–small cell lung cancer; PSP, progressive supranuclear palsy; PTM, post‐translational modification.

## 7 IN VITRO/EX VIVO METHODOLOGIES FOR INVESTIGATING MT DYNAMICS IN NEURODEGENERATION

Understanding MT dynamics is crucial for unraveling the mechanisms underlying neuronal dysfunction and degeneration. Biochemical assays, such as in vitro dynamic instability studies, allow precise measurements of MT growth and shrinkage rates under controlled conditions. Techniques such as mass spectrometry and proteomics have identified PTMs, such as acetylation and polyglutamylation, that regulate MT stability. Although these approaches provide detailed molecular insights, they lack the temporal resolution and systemic perspective offered by in vivo imaging techniques.^124^

Fluorescence microscopy, including advanced methods such as Total Internal Reflection Fluorescence (TIRF) and Stimulated Emission Depletion (STED), offers high spatial and temporal resolution, capturing real‐time polymerization and depolymerization events.^125^ In addition, cryo‐EM provides near‐atomic resolution of MT structures, revealing detailed insights into tubulin interactions and conformational changes.^126^ However, both methods are limited to in vitro or ex vivo studies and cannot penetrate deep tissues, making them unsuitable for systemic investigations, particularly in living human patients with neurodegenerative disease. In contrast, PET imaging offers a unique capability to provide real‐time, systemic, and quantitative assessments of MT dynamics in vivo.^13^ PET imaging can be integrated with complementary techniques such as microscopy for cellular‐level validation and proteomics for molecular insights, enabling a comprehensive understanding of MT behavior across different biological scales.

## 8 IN VIVO PET IMAGING OF MT INSTABILITY: A DIAGNOSTIC IMAGING TOOL

Despite its pivotal role in neuronal dysfunction and degeneration, MT instability has remained an underexplored target for in vivo imaging. This gap is largely due to the historical lack of imaging tools capable of quantifying MT dynamics in vivo.^127^ Recent advancements in PET radiotracers, particularly those targeting MTs, offer transformative potential for diagnosing neurodegenerative disorders and monitoring disease progression in real time. MT PET imaging has great potential to illuminate the many key roles that MT dysregulation plays in neurodegenerative processes.

### 8.1 Limitations of early MT radiotracers

MT dysfunction often manifests before clinical symptoms in neurodegenerative diseases, making it a promising early marker of neuronal pathology. However, the inability to non‐invasively image MT dynamics in the CNS has posed significant challenges. Notably, many early radiotracers were radiolabeled derivatives of MT‐stabilizing agents originally developed for cancer therapy. These included [^11^C]paclitaxel, [^18^F]fluoropaclitaxel, and [^11^C]docetaxel, which target the taxane binding site but exhibited limited brain uptake due to their susceptibility to efflux by BBB transporters such as P‐gp, MDR1, and BCRP.^128^ Similarly, [^11^C]colchicine and derivatives of 2‐methoxyestradiol and chelidonine showed some promise as MT‐binding agents but failed to demonstrate efficacy for CNS imaging in vivo.^129^ The development of [^11^C]MPC‐6827 marked a breakthrough in this field, addressing the limitations of earlier radiotracers with excellent BBB penetration.^14^

### 8.2 Breakthrough with [^11^C]MPC‐6827

[^11^C]MPC‐6827 is derived from MPC‐6827, a BBB‐penetrant MT‐targeting agent with high‐affinity binding to the β‐tubulin site (half maximal inhibitory concentration (IC_50_ )= 1.5 nM). MPC‐6827 has demonstrated suppression of tumor growth in various cancer models, is proven safe for use in human subjects, and has undergone multiple clinical trials for the treatment of glioblastoma cancer.^130^, ^131^ Kumar et al.^14^ utilized the exclusive CNS targeting properties of MPC‐6827 to develop [^11^C]MPC‐6827 as a potential MT PET imaging agent. The reliability of the automated radiosynthesis of [^11^C]MPC‐6827 was demonstrated by high specific activity and 100% reproducibility. The radioligand exhibited BBB penetration in mice and rats and was retained in the brain, consistent with its high‐affinity specific binding.

Although MPC‐6827 is pharmacologically active at therapeutic doses, the use of [^11^C]MPC‐6827 in PET imaging involves administration at tracer‐level mass doses (in the sub‐nanomolar range), typically in microcuries. These quantities are far below the thresholds required to exert any biological effects on MT dynamics. Thus, the radiotracer acts as a passive imaging agent and is not expected to perturb the underlying MT stability it is intended to measure.

In parallel, other MT‐targeting scaffolds have also been evaluated for CNS imaging potential. Notably, HD‐800, a selective colchicine‐site tubulin inhibitor, was identified with high binding affinity (IC_50_ = 4.3 nM in MDA‐MB‐435 cells; EC_50_ = 24 nM in A‐10 cells).^132^ The methoxy‐ and methylamine‐substituted derivatives of HD‐800 were amenable to [^11^C]‐labeling (O‐[^11^CH_3_] and N‐[^11^CH_3_]), enabling preliminary preclinical PET imaging studies.^133^ These tracers showed good BBB penetration, high brain‐to‐muscle ratios, and comparable specific binding to [^11^C]MPC‐6827 in mice. Likewise, WX‐MB‐18B (IC_50_ = 1.9 nM, IC_50_ = 0.45–0.99 nM), a structurally distinct MT inhibitor, also demonstrated favorable brain uptake and is suitable for [^11^C]‐radiolabeling.^134^ In addition, analogs amenable to [^18^F]‐labeling have been synthesized and evaluated in preclinical settings, although these remain in early stages of development and require further validation.^135^

#### 8.2.1 Mechanism and specificity

Building on the favorable imaging characteristics of [^11^C]MPC‐6827, its mechanistic specificity has been investigated using a series of in vitro and in vivo models to better understand its binding behavior under MT‐stabilizing and MT‐destabilizing conditions.

**FIGURE 3:**
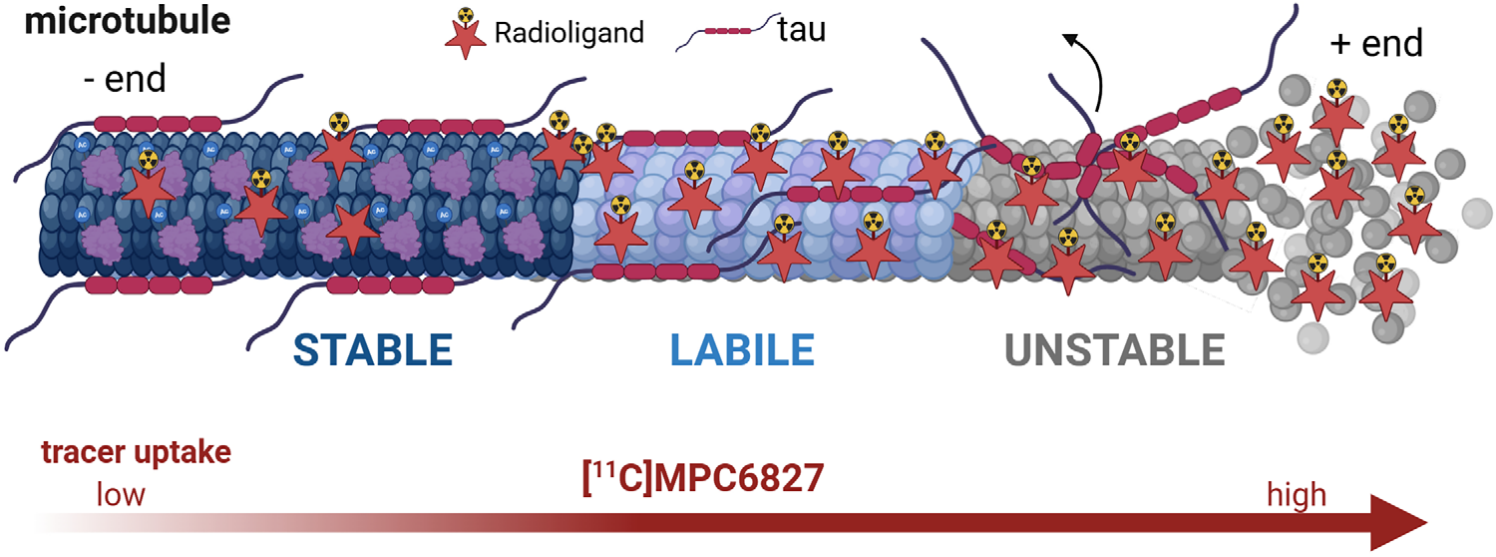
Schematic model representation of [^11^C]MPC‐6827′s preferential binding to destabilized microtubules (MTs). This illustration depicts the affinity profile of the positron emission tomography radiotracer [^11^C]MPC‐6827 along the dynamic spectrum of MT stability, ranging from stable to labile to fully unstable states. In the stable region, MTs are tightly packed and highly acetylated, with minimal [^11^C]MPC‐6827 binding (indicated by sparse red star symbols). As the MTs transition into the labile region, partial disassembly exposes more β‐tubulin binding sites, resulting in increased tracer binding. In the unstable region, extensive depolymerization and loss of structural integrity reveal abundant binding sites, leading to maximal [^11^C]MPC‐6827 uptake. The red gradient bar below the schematic visually represents the increase in [^11^C]MPC‐6827 uptake from stable to unstable MT conditions, supporting its mechanistic sensitivity to MT disassembly.

In vitro studies using SH‐SY5Y human neuroblastoma cells revealed differential uptake of [^11^C]MPC‐6827 under MT‐stabilizing and MT‐destabilizing conditions. Pharmacological stabilization with compounds such as EpoD and paclitaxel resulted in decreased radiotracer uptake, whereas destabilizing agents like mertansine and vinblastine significantly increased its retention. Cytoskeleton fractionation assays further confirmed the radiotracer's higher affinity toward free tubulin fractions under destabilized conditions, reinforcing its potential as a sensitive indicator of MT integrity.^127^, ^136^ Figure 3 provides a model representation of [^11^C]MPC‐6827 preferentially binding to destabilized MTs, illustrating its specificity and potential diagnostic utility.

The translational utility of [^11^C]MPC‐6827 was assessed in tau knockout (KO) mice, which lack tau's stabilizing influence on MTs. PET imaging studies demonstrated increased radiotracer uptake in tau KO mice compared to wild‐type controls, suggesting tau protein loss contributes to MT instability detectable by the tracer. This underscores the tracer's potential in investigating tau‐independent MT dysregulation beyond traditional tauopathy models.^127^

Further validation in rodent models of substance use disorder demonstrated reduced [^11^C]MPC‐6827 uptake following chronic exposure to alcohol and cocaine, suggesting drug‐induced stabilization of MTs over time. These findings highlight the tracer's ability to monitor dynamic changes in MT integrity in response to external stimuli, opening new avenues for substance abuse research.^136^, ^137^, ^138^

#### 8.2.2 Early AD detection in mice

**FIGURE 4:**
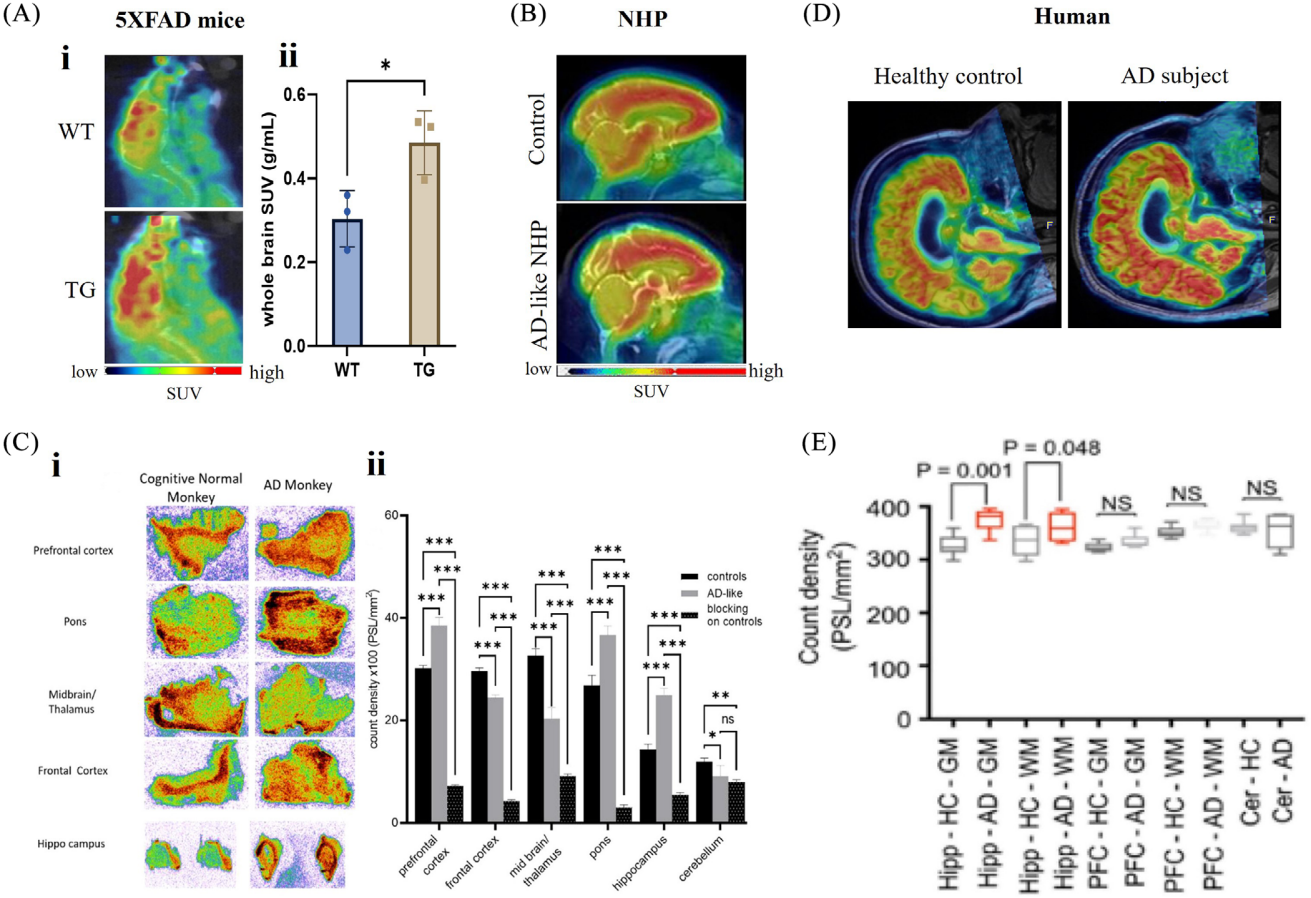
Positron emission tomography (PET) imaging and autoradiography evaluation of [^11^C]MPC‐6827 uptake in Alzheimer's disease (AD) models and human brain tissues. Damuka et al. (2024) demonstrated increased uptake of [^11^C]MPC‐6827 in 5xFAD mice compared to wild‐type (WT) controls, as shown by representative sagittal PET images of 12‐month‐old mice (A‐i), along with whole‐brain standardized uptake value (SUV) quantification (A‐ii). Bhoopal et al. (2023) presented representative PET/CT images illustrating [^11^C]MPC‐6827 brain uptake in control and AD‐like non‐human primates (NHPs) (B), as well as in vitro autoradiograms from an AD‐like NHP displaying regional binding of the tracer (C‐i), accompanied by quantitative comparison of regional uptake between control and AD‐like NHPs (C‐ii). Sai et al. (2024) presented representative PET/MRI images illustrating [^11^C]MPC‐6827 brain uptake in health control and AD subjects (D); Lindberg et al. (2021) reported preclinical characterization of [^11^C]MPC‐6827 in postmortem human brain tissues, revealing differences in total binding between gray matter and white matter across the hippocampus, prefrontal cortex, and cerebellum from healthy controls and AD patients (E).

Damuka et al. ^139^ explored the potential of [^11^C]MPC‐6827 for early detection of MT dysfunction in AD using longitudinal PET imaging in transgenic mouse models, including amyloid precursor protein/presenilin 1 (APP/PS1), PS19 transgenic mice (P301S), and five familial AD (5xFAD) (Figure 4A), which exhibit early Aβ and tau pathology. PET imaging at different disease stages revealed early increases in tracer uptake, concordant with MT destabilization prior to significant behavioral deficits. This suggests that [^11^C]MPC‐6827 could serve as an early biomarker for MT dysfunction, enabling timely intervention.

#### 8.2.3 NHP aging model insights

Damuka et al.,^140^ Lindberg et al.,^141^ and Bhoopal et al.^142^ examined a non‐human primate (NHP; vervet) aging model that naturally develops several hallmarks of AD with advancing age, using [^11^C]MPC‐6827 PET imaging (Figure 4B, C) as well as other AD biomarkers. The radiotracer displayed favorable pharmacokinetics and high uptake in neurodegeneration‐prone brain regions, with significant test–retest reliability. Tracer uptake inversely correlated with cerebrospinal fluid (CSF) Aβ42 levels, supporting the role of MT PET in tracking disease progression and treatment response. In vitro autoradiography experiments further showed that vervet brains with lower CSF Aβ42 exhibited higher [^11^C]MPC‐6827 uptake in prefrontal cortex, pons, and hippocampus compared to those with higher CSF Aβ42. In contrast, the uptake in midbrain/thalamus, frontal cortex, and cerebellum was slightly lower in comparison to control monkey brains. Together, these studies demonstrate the translational utility of [^11^C]MPC‐6827 PET in NHPs for linking MT dysfunction with established AD biomarkers, supporting its potential as a bridge between rodent models and human clinical studies.

#### 8.2.4 Clinical translation in AD

Autoradiography studies by Lindberg et al.^141^ in human post‐mortem AD and healthy brain tissues revealed significantly higher [^11^C]MPC‐6827 binding in hippocampal gray and white matter, with no significant differences in prefrontal cortex or cerebellum (Figure 4E). Sai et al.^143^ further evaluated the radiotracer in cognitively impaired and cognitively normal older adults, demonstrating higher uptake in the frontal cortex, hippocampus, and putamen in impaired subjects (Figure 4D). Uptake positively correlated with tau and Aβ PET markers and inversely with CSF Aβ^42^ levels, validating [^11^C]MPC‐6827 as a reliable biomarker for MT destabilization in human AD.

Beyond AD, [^11^C]MPC‐6827 holds promise for other neurodegenerative disorders. In ALS, where MT loss and axonal transport deficits are prominent, the tracer offers a means to quantify MT alterations and assess therapeutic interventions.^144^, ^145^ It may also provide insights into MT dysfunction in PD, HSP, HD, addiction, and radiation‐induced brain injury for broadening its translational imaging applications.^146^, ^147^ However, further clinical validation is necessary to determine whether disease‐specific regional uptake patterns can support differential diagnosis or whether MT imaging will be more broadly suited for monitoring progression and therapeutic response across neurodegenerative diseases.

## 9 CONCLUSIONS

MT instability represents a critical, yet underappreciated, early event in the pathogenesis of neurodegenerative diseases. Through this review, we highlight the central role of MT dynamics in maintaining neuronal structure and function, and how their disruption contributes to disease progression. Advances in molecular and structural biology have deepened our understanding of MT regulation, whereas recent developments in MT‐targeted PET imaging, particularly with [^11^C]MPC‐6827, have opened new avenues for non‐invasive diagnosis and therapeutic monitoring. Cross‐species validation from rodent models to non‐human primates and human studies underscores the translational promise of MT imaging. As research continues to unravel the molecular mechanisms driving MT dysfunction, integrating biochemical, imaging, and therapeutic strategies will be key to developing early diagnostic tools and disease‐modifying interventions. Targeting MT instability holds significant potential to shift the current paradigm in neurodegenerative disease research from reactive treatment to proactive intervention.

The refinement of MT‐specific radiotracers, alongside longitudinal imaging, holds promise for identifying preclinical disease stages and stratifying at‐risk populations. When integrated with established biomarkers like tau, amyloid, and neuroinflammation, MT imaging can enhance diagnostic precision and offer a more complete view of disease progression. This approach supports earlier intervention, improved clinical trial design, and more personalized care strategies for individuals affected by or at risk for AD.

## CONFLICT OF INTEREST STATEMENT

The authors declare no conflicts of interest. Any author disclosures are available in the Supporting Information.

## Supporting information



Supporting Information
